# A single vaccination with an inactivated bovine respiratory syncytial virus vaccine primes the cellular immune response in calves with maternal antibody

**DOI:** 10.1186/1746-6148-6-2

**Published:** 2010-01-11

**Authors:** Mirjam TW van der Sluijs, Eva M Kuhn, Birgit Makoschey

**Affiliations:** 1 Virological R&D Department, Intervet International b.v., Wim de Körverstraat 35, NL-5831 AN, Boxmeer, the Netherlands; 2 Quality Management Biologicals Department, Intervet International b.v., Wim de Körverstraat 35, NL-5831 AN, Boxmeer, the Netherlands; 3 Global Ruminant Business Unit, Intervet International b.v., Wim de Körverstraat 35, NL-5831 AN, Boxmeer, the Netherlands

## Abstract

**Background:**

The efficacy of a single dose of an inactivated bovine respiratory syncytial virus (BRSV) - Parainfluenaza type 3 (PI3) - *Mannheimia haemolytica *(*Mh*) combination vaccine, in calves positive for maternal antibodies, was established in a BRSV infection study.

**Results:**

As expected the single vaccination did not have any effect on the decline of BRSV-specific neutralising or ELISA antibody. The cellular immune system was however primed by the vaccination. In the vaccinated group virus excretion with nasal discharge was reduced, less virus could be re-isolated from lung tissues and the lungs were less affected.

**Conclusions:**

These results indicate that a single vaccination with an inactivated BRSV vaccine was able to break through the maternal immunity and induce partial protection in very young calves. It can be speculated that the level and duration of protection will improve after the second dose of vaccine is administered. A two-dose basic vaccination schedule is recommended under field conditions.

## Background

The bovine respiratory syncytial virus (BRSV) has been established as an important viral component in the bovine respiratory disease complex (BRDC) [1]. The virus can act synergistically with other viruses such as the bovine parainfluenza type 3 virus (PI3) or bacteria such as *Mannheimia haemolytica *(*Mh*), *Pasteurella multocida, Haemophilus somnus *and *Mycoplasma bovis *to cause pneumonia [2]. BRSV also fulfils the Koch's postulates to be recognised as an aetiological agent of pneumonia in calves. Respiratory disease is however often milder under laboratory conditions than under field conditions [3-6].

Outbreaks of BRSV associated pneumonia typically recur every year [7] and most farms are affected. The mechanisms for the persistence of the virus in a farm are not well understood [8]. Based on sequence divergence among BRSV isolated during recurrent outbreaks in closed herds, (re)introduction of the virus into the herd prior to each new outbreak is the most likely explanation [9]. Airborne transmission of BRSV has been demonstrated under experimental conditions [10].

Different approaches have been followed to develop safe and efficacious BRSV vaccines including inactivated vaccines [11,12], (genetically) modified live vaccines [11,13], subunit vaccines [14], DNA vaccines [15] and vector vaccines [16,17]. Today inactivated and modified live BRSV vaccines are commercially available, most of them as combination vaccine with other antigens related to the BRDC.

The basic vaccination schedule for inactivated BRSV vaccines consist of two doses of 5 ml each, applied by subcutaneous injections, at an interval of 3 to 4 weeks. Remarkably most cases of severe BRSV are observed in calves aged 1-3 months, at which time they still possess maternal antibodies [18]. In order to provide efficacious protection against BRSV at this very young age it is essential that vaccines are able to overcome the interference of maternal antibodies and confer protection shortly after vaccination. Due to the high risk of BRSV associated pneumonia in the very young age group, it is also of great value to evaluate the level of immunity induced by the first vaccination before completion of the full primary course has taken place. The inactivated BRSV-PI3-*Mh *combination vaccine used in the study reported here has previously been shown to reduce the severity of clinical signs and virus shedding in two week-old MDA (+) calves that had received a primary course of two vaccinations four weeks apart. The calves were challenged with BRSV 3 weeks after the second vaccination. Moreover, in a previous study with the same vaccine, the efficacy in terms of antibody response and clinical protection has been established after application of a single dose of the vaccine to 4-5 week old calves with maternal antibodies [19]. In the study reported here two week old calves with maternal antibodies were vaccinated with a single dose, antibody levels as well as cellular immunity was measured. As it was expected that the maternal antibodies would interfere with the induction of clinical signs, lung pathology was used as a parameter to evaluate protection.

## Methods

### Animals

Ten Holstein crossbred calves were included in the study. They were removed from the dam directly after birth in order to prevent infection with BRSV or other pathogens. The calves were fed pre-collected colostrums with high antibody titres against BRSV in order to obtain similar antibody titres in all calves. The animals were identified by means of numbered ear tags. They were ranked by age and then allocated, on an alternating basis, to one of the two treatment groups in order to obtain the same average age for both groups. The complete study was undertaken in a blinded manner.

From the week before vaccination and onwards the calves were housed, separated by group, in two different rooms of an isolation unit. Strict barrier conditions between the two groups were maintained by means of individual filtered air supply to each room, sealed doors and by staff changing cloths and showering between the rooms.

One calf allocated to the control group had to be euthanised because of a persistent inflammation of the carpal joint before challenge.

All procedures were approved upfront by the Animal Care and Use Committee according to the Dutch animal welfare regulations.

### Vaccination

A commercial batch of an inactivated combined BRSV, PI3 and Mh vaccine with alhydrogel and Quil A as adjuvants (Bovilis Bovipast RSP, Intervet/Schering-Plough Animal Health) was used. Five calves were given a single dose of 5 ml by the subcutaneous route into the neck. A second group of 5 calves was not vaccinated. The average age of the calves at the time of vaccination was two weeks.

### Challenge

Four weeks after they had been vaccinated, all calves were challenged by aerosolisation with a wild-type strain of BRSV (CA-1, kindly provided by L. Gershwin) as described earlier [13]. The challenge dose was 10^5.9 ^TCID_50 _in a 10 ml volume.

### Assessment of clinical signs

From two days prior to challenge until the end of the study the calves were observed daily for clinical reactions and the body temperature was recorded. Special attention was paid to signs of respiratory disease such as coughing - spontaneous and stimulated (by palpation of the larynx), nasal or ocular discharge and respiratory rate.

### Serology

To determine the calves' antibody response to vaccination and challenge, blood samples were taken at vaccination (day 0), just before challenge (day 28) and one week later (day 35). Serum samples were assayed for BRSV specific antibody by serum neutralising (VN) antibody and ELISA antibody.

The VN test was performed according to the end point dilution method in a microtitre format. Two-fold serial dilutions of the serum samples were incubated with a fixed amount of BRSV prior to the addition of Vero cells. After 5-7 days of culture, the plates were read for BRSV specific cytopathic effects. The VN titres were the reciprocal value of the highest dilution in which complete neutralisation of the virus was obtained. They were expressed as log_2_.

An in-house BRSV blocking ELISA was performed. Plates were first coated with a BRSV specific monoclonal antibody and secondly with BRSV antigen. After appropriate washing steps, dilution series of the sera were incubated followed by the incubation of a second biotinylated monoclonal antibody. Avidin-peroxidase complexes were allowed to bind to the biotin. A standard colour reaction using 3,3';5,5'tetramethylbenzidine as substrate was performed to quantitate the amount of bound second antibody. Antibody titres were calculated from the blocking percentage.

### Cellular immune response

A lymphocyte activation assay was performed on heparinised blood samples taken at days 0, 28 and 35. In 24-well plates heparinised blood samples (sample volume: 1 ml) were stimulated with Concanavalin A (Sigma Aldrich, 100 μl/ml blood) or lysate of BRSV infected MDBK cells. Negative control samples were treated similarly with culture medium or lysate of uninfected MDBK cells. After two days of incubation at 37°C Interferon γ (IFN γ) production was measured using a commercial ELISA (Bovine IFN γ EASIA, Biosource Europe S.A.) according to the manufacturer's recommendations.

### Nasal shedding of challenge virus

From two days prior to challenge until the end of the study nasal swab samples were taken daily from all calves by means of swab sticks containing a sterile cotton plug. Separate swabs were used for each animal. The swabs were gently rotated up and down the sides of both nasal cavities several times. The swabs were then inserted into a tube containing 1 ml of sterile phosphate buffered saline medium. The liquid was transferred into suitable containers and stored at temperatures below -70°C until tested.

### Post mortem examination

At seven or 10 days after challenge the calves were sacrificed. The lungs were inspected macroscopically and representative samples of organs showing any changes were taken. The samples were fixed with formalin and then processed into paraffin embedded sections. These were stained with haematoxylin and eosin and investigated microscopically.

Additional samples for virus isolation were taken from nasopharyngeal mucosa, tonsils, submandibular lymph nodes, each lung lobe and mediastinal and bronchial lymph nodes.

Tracheobroncheal lung washes were performed as follows. Both lungs were excised and flushed with 300 ml of saline. The liquid was recovered and stored at temperatures below -70°C until tested.

### Titration of nasal swab samples and lung wash fluid for BRSV

The BRSV titre in nasal swab samples and lung wash fluid was determined according to the end point dilution method in a microtitre format. Five-fold serial dilutions of the samples were incubated on bovine embryo lung cells. Infection was evaluated by the development of BRSV specific cytopathic effects after 5-7 days of culture. Each sample was tested in quadruplicates.

### Testing of tissue samples for BRSV

Homogenized samples were incubated in four-fold on a monolayer of Vero cells in 24-well plates. After 5-7 days of culture supernatants of the cultures were transferred onto fresh monolayers of Vero cells. The monolayers after the first and the second passage were read for BRSV specific cytopathic effects.

### Statistical evaluation

The results were summarised using frequency tables. The Wilcoxon two-sample test was performed to compare: maximum titres in nasal swab samples, days of virus excretion and extinction values in the INFγ ELISA. 

The above statistical tests were performed at 5% level of significance. The data analysis was done using SAS software, Version 8.2. Copyright^© ^[1999-2001] by SAS Institute Inc., Cary, NC, USA.

## Results

### Clinical signs and body temperatures

Only minor clinical signs were observed. There were no increased respiratory rates; in fact the only clinical sign of respiratory disease was coughing of individual animals. One vaccinated animal showed stimulated coughing, during examination, at 6 and 7 days after challenge. Another vaccinated and one unvaccinated animal coughed spontaneously on days 8 through 10.

Throughout the experiment none of the calves developed a fever (body temperature above 39.5°C).

### BRSV specific immune reaction

#### BRSV specific antibody response

All calves had moderate to high antibody titres at the start of the study (individual data not shown). The average VN and ELISA antibody titres at the time of vaccination in the vaccinated group were 9.6 log_2 _and 8.2 log_2 _(see table 1). These values are very similar to the average titres measured at the same time for the control animals (9.3 log_2 _and 8.3 log_2 _for VN and ELISA respectively). At the time of challenge (t = 28 days) and one week later (t = 35 days) the titres were at the same level or had dropped slightly for both groups.

**Table 1:** BRSV specific humoral immune response after vaccination and challenge

Parameter	VN antibody			ELISA antibody		
Time [days]◆	0	28	35	0	28	35

Treatment group:						

Vaccinated	9.6	8.0	7.6	8.2	6.5	6.5

Unvaccinated	9.3	8.4	7.5	8.3	6.6	6.5

◆ Calves were infected intranasally with live BRSV 28 days after vaccination with one dose of the inactivated BRSV-PI3-*Mh *combination vaccine.

#### BRSV specific cellular immune response

No cellular immune response, as determined in the lymphocyte stimulation assay, was detected at the time of vaccination (see table 2). At the time of challenge one out of five vaccinated animals were positive, all unvaccinated animals were negative.

**Table 2:** BRSV specific cellular immune response (extinction values of IFN γ ELISA on stimulated whole blood samples after vaccination and challenge)

Time [days]◆:		0	28	35
**Treatment group**	**Animal**			

Vaccinated	V1	0.057*	0.185	**0.736**
	
	V2	0.056	**0.267**	**0.476**
	
	V3	0.054		**0.223**
	
	V4	0.051	0.121	**0.542**
	
	V5	0.052	0.054	**0.408**

	Average^#^	0.054^a^	0.157^a^	**0.477**^a^

Unvaccinated	C1	0.055	0.059	0.099
	
	C2	0.052	0.060	**0.214**
	
	C3	0.056	0.055	0.106
	
	C4	0.057	0.060	0.067

	Average	0.055^a^	0.059^a^	0.121^b^

◆ Calves were infected intranasally with live BRSV 28 days after vaccination with one dose of the inactivated BRSV-PI3-*Mh *combination vaccine.

*According to the information of the manufacturer of the assay, values above the cut-off level (0.207) have to be interpreted as positive - indicated by the bold letter type

^# ^Average values with different superscript letters are significantly different (p < 0.05) from each other.

One week after the challenge IFN γ production was measured in stimulated blood samples of four out of five vaccinated and one out of four unvaccinated control calves. The difference between the two groups was statistically significant (p < 0.05, Wilcoxon two-sample test).

### Virus re-isolation from nasal swab samples

From day 4 to day 7 after infection BRSV could be re-isolated from nasal swab samples from three out of four unvaccinated animals. (table 3). Only two out of five vaccinated animals shed detectable but low levels of BRSV with nasal discharge on day 6 post-infection. The maximum titres and the number of days when virus was excreted was significantly lower in the vaccinated group as compared to the control group (p < 0.05, Wilcoxon two sample test.)

**Table 3:** Individual BRSV infection titres [log_10_**TCID**_50_**] in nasal swab samples in vaccinated and control calves after intranasal challenge with live BRSV**

Treatment group	Animal	Days after infection										
		
		0	1	2	3	4	5	6	7	8	9	10
Vaccinated	V1	-	-	-	-	-	-	-	-	n.s.	n.s.	n.s.
	
	V2	-	-	-	-	-	-	2.7	-	n.s.	n.s.	n.s.
	
	V3	-	-	-	-	-	-	-	-	n.s.	n.s.	n.s.
	
	V4	-	-	-	-	-	-	2.0	-	-	-	-
	
	V5	-	-	-	-	-	-	-	-	-	-	-

Control	C1	-	-	-	-	-	-	-	-	n.s.	n.s.	n.s.
	
	C2	-	-	-	-	-	2.0	3.9	2.0	n.s.	n.s.	n.s.
	
	C3	-	-	-	-	-	-	3.6	3.4	3.6	-	-
	
	C4	-	-	-	-	3.2	-	3.8	-	-	-	-

-: sample negative (titre below detection limit of 2.0 log_10_TCID_50 _/ml)

n.s. no sample (animal sacrificed 7 days after infection)

### Gross and microscopic pathology findings

Macroscopic pathological findings differed substantially between the animals No lung lesions were observed in the lungs of two vaccinated and one unvaccinated animal. Only minor lesions were present in the lungs of two vaccinated and two unvaccinated animals.

The lung of the fifth vaccinated animal showed dark red consolidation of 10-25% of the medial and apical lung lobes. In contrast all lung lobes of the fourth unvaccinated animal were affected and 50% of the right apical lobe and 80% of the medial lobe were consolidated.

BRSV non-specific microscopic lesions were present in two vaccinated and three control animals. Syncytial cells, which are characteristic for BRSV infection, were present in two vaccinated animals and suspected in one control animal.

### Isolation of BRSV from tissue samples and lung wash fluid

BRSV could be re-isolated from tissue samples out of the respiratory tract in four out of five vaccinated and all unvaccinated animals (see table 4). The number of positive samples was higher in the unvaccinated group (average 4.5) than in the vaccinated (2.0).

**Table 4:** BRSV isolation from tissue samples and lung wash fluid of vaccinated and control animals after intranasal infection with BRSV

Treatment group	Animal	Post mortem on dpi^a^	Number tissue sample	BRSV titre in lung wash log_10 _TCID_50_/ml
Vaccinated	V1	7	1	-
	
	V2	7	5	-
	
	V3	7	0	4.5
	
	V4	10	1	-
	
	V5	10	3	-

Control	C1	7	6	-
	
	C2	7	5	5.4
	
	C3	10	4	-
	
	C4	10	3	-

^a◆ ^dpi: days post infection

The lung wash fluid of one vaccinated and one unvaccinated animal, both killed 7 days after infection, was found positive for BRSV. All other samples were negative.

## Discussion

The clinical signs observed after intranasal challenge with virulent BRSV were rather mild in the control animals and therefore no conclusion on clinical protection can be drawn from this study. The difficulty of reproducing BRSV associated disease under experimental conditions has been reported previously [3-6]. In the study reported here calves with maternally derived antibodies were used and the challenge was performed as early as 6 weeks of age. At that point maternal antibody levels were still moderate to high. These maternal antibodies are likely to interfere with the BRSV infection as reported by Kimman and colleagues [18]. For this reason BRSV vaccination-challenge studies reported in the literature were often either performed in older animals [19,20] or colostrum deprived calves [21]. or the challenge was performed later after vaccination [22,23], alternatively a combination of these different approaches was applied. However an early challenge of young calves with moderate to high maternally derived immunity, as described in this study, reflects the field situation. Under these conditions it is very difficult but also very important for a BRSV vaccine to provide protection.

The vaccinated animals did not develop clinical signs other than provoked or spontaneous coughing in one animal. This observation is noteworthy since vaccine induced exacerbation of BRSV infections has been reported from field observations [24] and reproduced in the laboratory [25]. The pathophysiology of the disease in cattle was related to formalin inactivated BRSV. This is analogous to reports on human RSV enhancement after vaccination with formalin-inactivated RSV vaccine. The hypothesis is however not in line with the results of Mohanty and co-workers [26]. The vaccine used in this study also contained formalin-inactivated BRSV. The number of animals used here might be too low to allow general conclusions, however our results confirm earlier findings from laboratory studies [12,19] and unpublished field data that the vaccine does not enhance BRSV disease severity in naturally infected cattle.

In this experiment a single dose of the inactivated BRSV-PI3-*Mh *combination vaccine did not have any effect on the decline of BRSV-specific neutralising or ELISA antibody. This result was expected and is in line with other reports [19,20]. The role of different types of antibodies in protection against BRSV has been discussed in the literature [27]. Therefore, in this study, VN titres and ELISA titres were determined. However both types of antibody titres, VN and ELISA, followed the same profile e.g. animals with high VN titres also had high ELISA titres and vice versa. This finding indicates that the colostrums used to feed the calves were obtained from mothers with a similar history of BRSV vaccination and/or infection.

Earlier studies with the same vaccine have shown a clear booster response when the animals were given a second dose of vaccine [20] or infected with BRSV 3 weeks after a single dose of the vaccine [19]. In this study calves were euthanised for post mortem examination at 7 or 10 days post infection, therefore no booster antibody response could be detected but it can be speculated that the animals would have reacted with a booster response.

A very interesting result in this study was the priming of the cellular immune response by a single dose of vaccine in the absence of a measurable antibody response. Kerkhofs and colleagues [20] also reported that the cellular immune response, determined by lymphoproliferation, was induced by the same inactivated BRSV vaccine. When discussed in the literature, the role of CD8 cells in BRSV infection is very controversial. While it has been reported that CD8 responses of animals vaccinated with a different inactivated BRSV vaccine did not protect against immunopathology [28], these immune cells seem to play a key role in the control of infection in calves [29]. This is consistent with the reduction of virus excretion with nasal discharge in vaccinated animals when compared to control animals in the study reported here. This result can be considered a key finding in the study, as a reduction in the excretion of challenge virus i) reflects the replication of the virus indicating that the vaccine is protective and ii) is of practical relevance because reduced virus shedding will also limit the risk of infection for in-contact animals.

Post mortem examinations were performed on two different days (7 or 10 days after infection) as it was expected that hardly any BRSV virus would be detectable more than 1 week after infection, while lung pathology would typically develop around 10 days after infection. In general the results of BRSV re-isolation and lung pathology in this study were in line with these expectations.

BRSV was re-isolated from samples of different organs from the respiratory tract of all control animals and from four out of five vaccinated animals. The overall number of positive samples was lower in the vaccinated group indicating that BRSV replication was reduced after vaccination.

This observation was also reflected by the lung pathology. Macroscopic and histological findings in vaccinated animals were minor and not comparable with the findings in lung sections of BRSV infected unvaccinated animals. From the results obtained it cannot be excluded that other pathogenic agents like *Mh *were also involved. The vaccine also contains *Mh *antigen and therefore also protects against this pathogen.

In general, more virus could be isolated from the animals euthanised seven days after infection while lung pathology seemed to be more severe in the animals euthanised at 10 days after infection. It has been reported earlier that lung pathology in BRSV infection is the consequence of immuno-pathological events rather that direct damage of the virus to the tissues [29].

## Conclusion

From the results described, it can be concluded that, a single vaccination with an inactivated BRSV vaccine was able to break through the maternal immunity and induce partial protection in very young calves. It can be speculated that the level and duration of the protection will improve once a second dose of vaccine is given. Therefore under field conditions the recommended vaccination schedule of two doses must be administered.

## Competing interests

All authors are employees of Intervet/Schering-Plough Animal Health, the company that markets the vaccine that has been used in the studies reported herein. The article processing charge is payed by Intervet/Schering-Plough Animal Health.

## Authors' contributions

MvdS and BM designed the experiments, MvdS and EK performed the experiment, MvdS, EK and BM analyzed the data. BM drafted the manuscript. All authors read and approved the final manuscript.
